# Surface Analysis of Wire-Electrical-Discharge-Machining-Processed Shape-Memory Alloys

**DOI:** 10.3390/ma13030530

**Published:** 2020-01-22

**Authors:** Rakesh Chaudhari, Jay J. Vora, Vivek Patel, L. N. López de Lacalle, D. M. Parikh

**Affiliations:** 1 Department of Mechanical Engineering, School of Technology, Pandit Deendayal Petroleum University, Raisan, Gandhinagar 382007, India; chaudharirakesh5@gmail.com (R.C.); profvvp@yahoo.com (V.P.); 2 School of Material Science and Engineering, Northwestern Polytechnical University, Xi’an 710129, China; 3 Department of Mechanical Engineering, University of the Basque Country, Escuela Superior de Ingenieros Alameda de Urquijo s/n., 48013 Bilbao, Spain; norberto.lacalle@ehu.eus; 4 Department of Industrial Engineering, School of Technology, Pandit Deendayal Petroleum University, Raisan, Gandhinagar 382007, India; dm.parikh@sot.pdpu.ac.in

**Keywords:** shape-memory alloy, WEDM, superelasticnitinol, surface integrity, surface roughness, SEM

## Abstract

Shape-memory alloys such as nitinol are gaining popularity as advanced materials in the aerospace, medical, and automobile sectors. However, nitinol is a difficult-to-cut material because of its versatile specific properties such as the shape-memory effect, superelasticity, high specific strength, high wear and corrosion resistance, and severe strain hardening. Anunconventional machining process like wire-electrical-discharge-machining (WEDM) can be effectively and efficiently used for the machining of such alloys, although the WEDM-induced surface integrity of nitinol hassignificant impact on material performance. Therefore, this work investigated the surface integrity of WEDM-processed nitinol samples using digital microscopy imaging, scanning electron microscopy (SEM), and energy-dispersive X-ray (EDX) analysis. Three-dimensional analysis of the surfaces was carried out in two different patterns (along the periphery and the vertical plane of the machined surface) andrevealed that surface roughness was maximalat the point where the surface was largely exposed to the WEDM dielectric fluid. To attain the desired surface roughness, appropriate discharge energy is required that, in turn, requires the appropriate parameter settings of the WEDM process. Different SEM image analyses showed a reduction in microcracks and pores, and in globule-density size at optimized parameters. EDX analysis revealed the absence of wire material on the machined surface

## 1. Introduction

Shape-memory alloys (SMAs) have been extensively used in the biomedical, aerospace, automobile, robotics fields, and in some other important industrial applications (such as eyeglass frames, cellular-phone antennas, and automotive devices) because of their shape-memory effect, high wear resistance, superelasticity, high corrosion resistance, biocompatibility, and high actuation strain [1,2,3]. SMAs are available in a wide range, such as Au–Cd, Cu–Zn, Cu–Sn, Cu–Al–Ni, Ti–Nb, and Ni–Ti [1]. Nickel titanium SMAs, popularly known as nitinol, have wide applications in various industries such as min actuators, micromechanical systems, automation and control, aerospace robotics, heating and ventilation, and biomedicine owing to their desirable properties such as high corrosion and wear resistance, mechanical simplicity and compactness, and maximal recoverable strain [1,3,4,5]. However, nitinol SMAs are popular for biomedical applications because of their higher shape-memory strain, good biocompatibility, and the prevention of chemicallyinduced illnesses [3,6,7,8,9]. With the application of heat, they regain their shape and size. Materials with superelastic and shape-memory effects are two differentSMA categories. The particular material is said to exhibit a superelastic effect if the shape-recovery temperature is below room temperature; if it is above room temperature, it produces a shape-memory effect [10]. However, the conventional machining of nitinol is challenging due to its superelasticity, high ductility, high wear and corrosion resistance, and severe strain hardening [11,12,13]. Researchers reported problems of conventional machining processes for shape-memory alloys [14,15]. K. Weinert and V. Petzoldt [14] concluded that the machining of NiTi-based alloys is complex using conventional techniques like turning, drilling, and deep hole drilling. Poor chip breaking, tool wear rate, and burr formation have been observed through conventional SMA machining techniques. High viscosity, toughness, severe strain hardening, and poor surface finishes are some of the difficulties observed by SMA researchers using conventional machining techniques [14,16]. To overcome these defects, wire-electrical-discharge machining (WEDM) is one of the best suitable techniques of nonconventional processesthatcan machine any electrically conductive material regardless of hardness and toughness [17,18,19,20,21]. The WEDM process works on the principle ofmaterial erosion due to the thermoelectric effect thatcan be achieved by controlled spark erosion between workpiece and travelling wire. This process involves a high number of input process parameters that need to be controlled to obtain good surface quality, and subsequent metallurgical and mechanical properties. Manjaiah et al. [22] simultaneously optimized the multiple output responses for NiTi SMAs using the WEDM process. Their results revealed that pulse-on time (T_on_) and pulse-off time (T_off_) highlighted a significant effect on all output variables. In another study, Chaudhari et al. [10] concluded that T_on_, T_off_, and current are the main influencing factors for the response variables of the material-removal rate(MRR), surface roughness (SR), and microhardness for the WEDMof nickel-based alloys. Soni et al. [23] explored the WEDM of Ti50Ni40Co10 SMA with the output variables of MRR and SR. Increasing values of MRR and SR were reported with increasing T_on_, while both values decreased with increasing T_off_ and servo voltage. Researchers used brass and copper wires as electrodes, and studied their effect on the surface integrity of amachinedSMA surface [19,20]. To the best of the authors’ knowledge, no study has been reported on the surface analysis of the machined surface of nitinol SMAs through the WEDM process using molybdenum wire. Very few studies were reported regarding the surface analysis of nitinol SMAs machined through the WEDM process. 

Recently, we demonstrated aparametric optimization study ofWEDM process parameters for the machining of nitinol shape-memory alloys using a heat-transfer-search algorithm [20]. Pulse-on time, pulse-off time, and current werethe selected important process parameters with the output responses of MRR, SR, and microhardness (MH). In the current study, surface analysis of the material that was machined at the optimalprocess-parameter setting is reported. From adetailed literature survey, it was found that the majority of research workwas carried out and concentrated on the parametric optimization of shape-memory alloys, but studies on surface integrity after WEDM for shape-memory alloys are rarely reported. In the current study, surface morphology, phase analysis, and elemental composition after WEDM using scanning electron microscope (SEM) and energy dispersive X-ray analysis (EDX) arediscussed. Thisstudy provides substantial input to end users working theWEDM of superelastic SMAs.

## 2. Materials and Methods 

**Figure 1:**
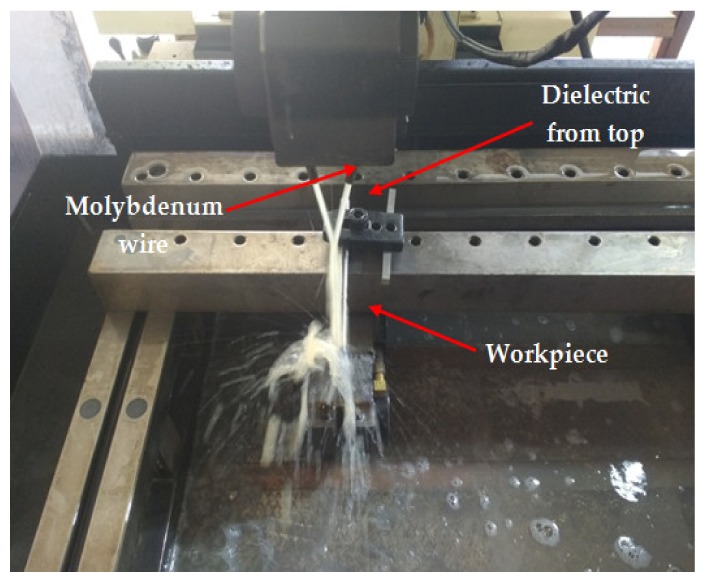
Representation of wire-electrical-discharge-machining(WEDM) process with flushing mechanism.

In the current study, the surface integrity of the machined surface of anitinol shape-memory alloy was carried out by using the WEDM process. Table 1 shows the chemical composition of the Ni55.8Ti work material used in the current study. A nitinol rod of 8 mmdiameterwasused to machine samples for surface analysis. In apast study, the influence of cutting parameters on output responses wasexamined by cutting sample sizes of 1.5 mm each thatweresuitable for measuringall responses. The same size of 1.5 mm was preferred in the current study. It wasalready concluded that T_on_, T_off_, and current were the three most important process parameters that were chosen for machining. The experiment was conducted on a Concord WEDM machine DK7732 with dielectric fluid as EDM oil. Reusable molybdenum wire with a diameter of 0.8 mm was used as a tool electrode. A side-flushing mechanism (from top) was used in the current study during the WEDM process, as shown in Figure 1.

Thethree-dimensional (3D) superdepth digital microscope is an accurate and precise instrument that has a noncontact-type probe. Hence, a 3D superdepth digital microscope (Keyence VHX-600, China) wasused to measure the surface morphology of the WEDM-machined sample at various locations. A similar kind of the 3D analysis of a friction-stir-process Mg alloy wasrecently reported [24]. The arithmetic average-roughness (Ra) value was used to record the SR of the machined sample. Surface finish was measured on a 3D image of the surface at different locations of the material. To ensure a smooth surface for processing, the base material was ground. For surface analysis, the material sample was mechanically polished and etched (14 mL HNO_3_ + 4 mL HF + 82 mL H_2_O). A VEGA TESCAN-make scanning electron microscope (Vega Teskan, India) was used for surface morphology. The working distance between electron gun and workpiece surface was kept at 26.89 mm with measuring energy of 20 KV. Energy dispersive X-ray analysis (Vega Teskan, India) was carried out by connecting the instrument with Oxford software with anSEM machine. 

## 3. Results and Discussion

### 3.1. Surface-Roughness Analysis

**Figure 2:**
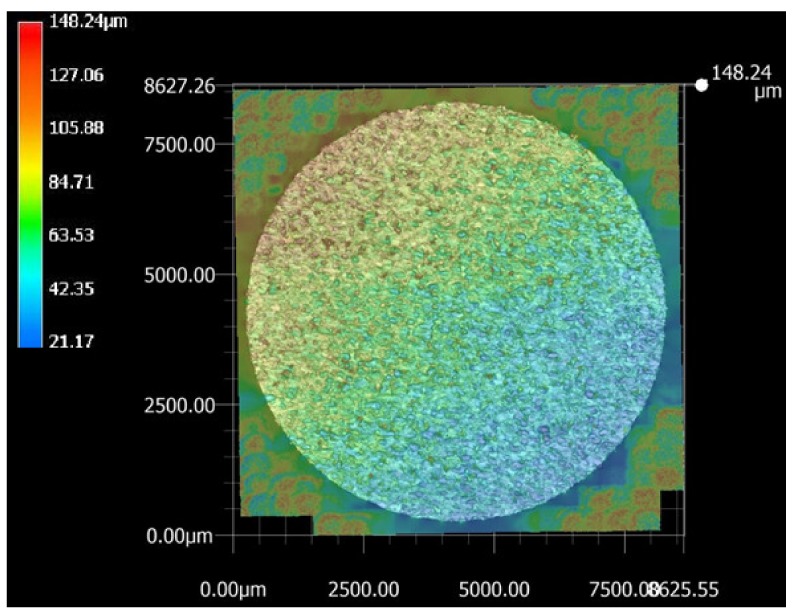
Top surface 3-D profie representing SR values

**Figure 3:**
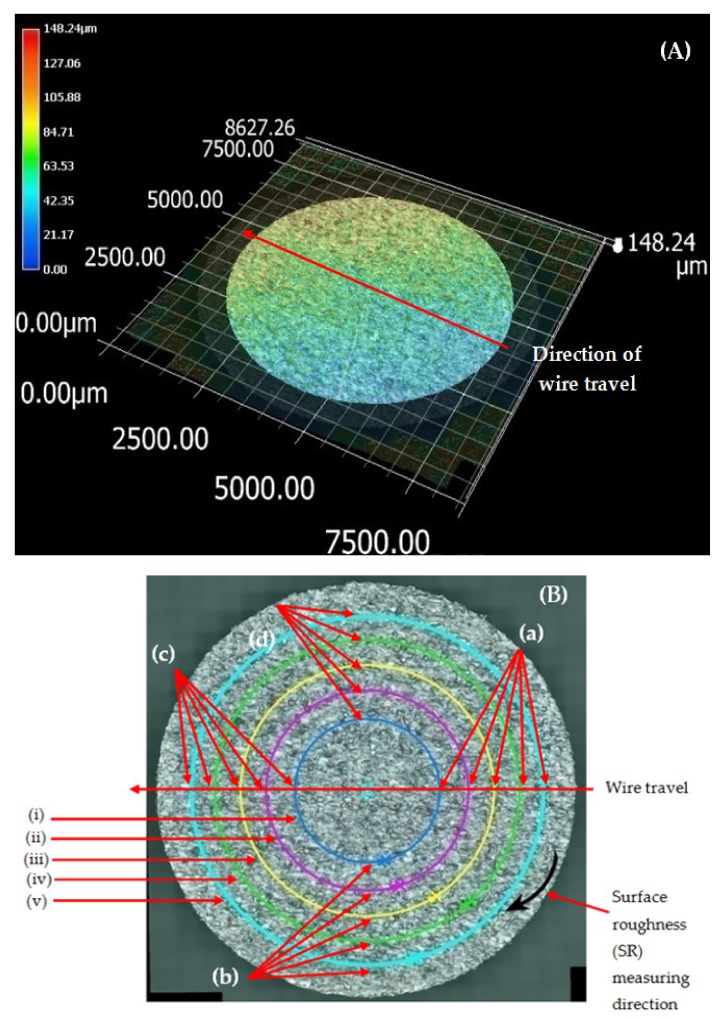
(**A**) Wire-travel direction; (**B**)Surface analysis for sample at outer surface.

**Figure 4:**
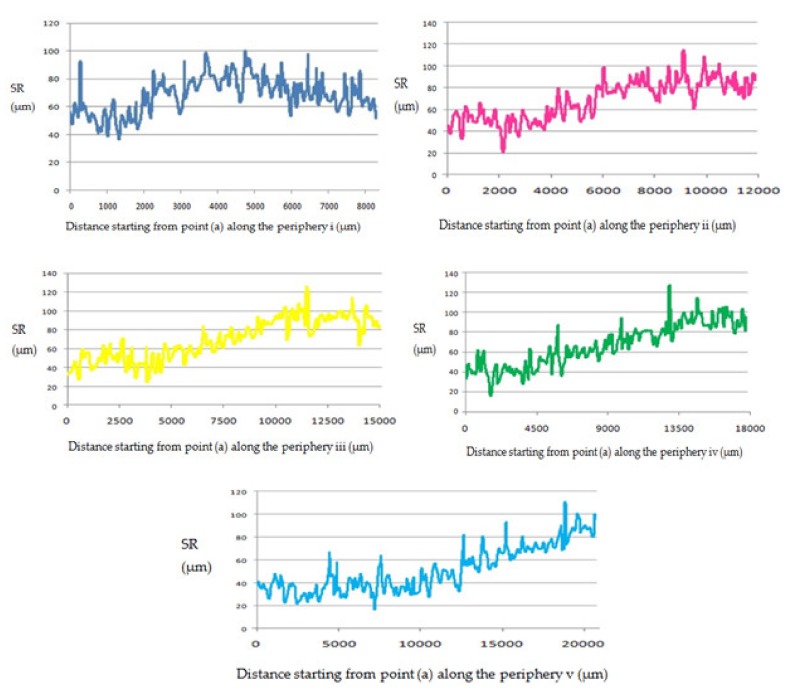
Roughness measurement along periphery.

**Figure 5:**
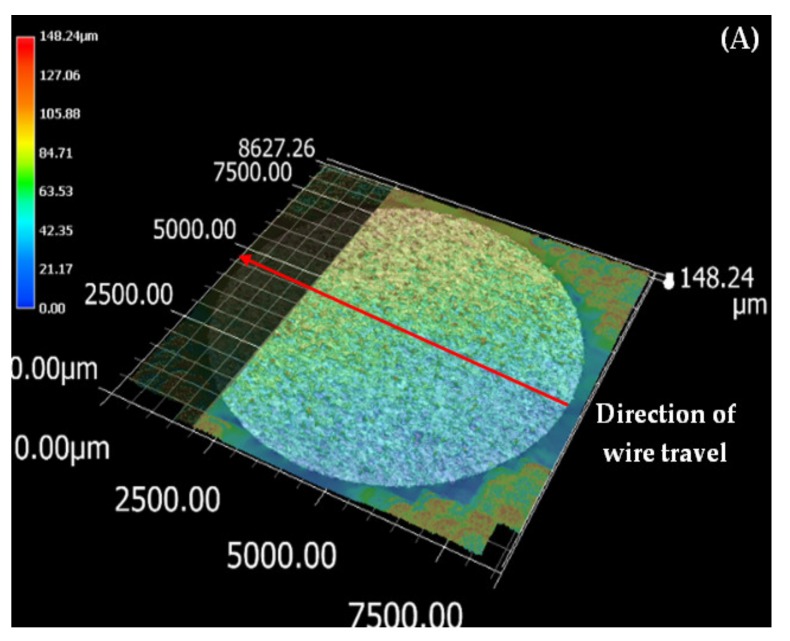

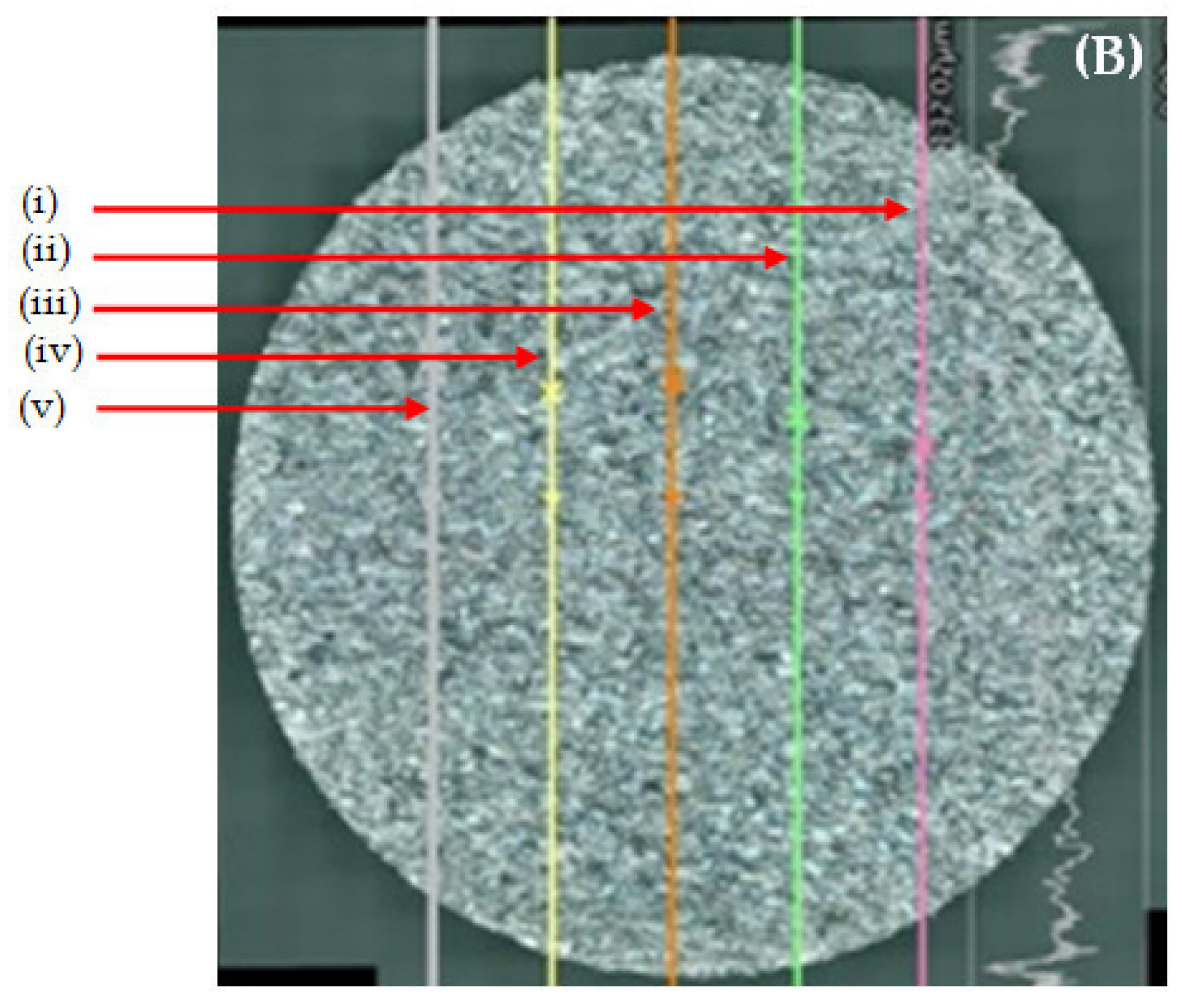
(**A**) Wire-travel direction; (**B**) surface analysis for sample at outer surface.

**Figure 6:**
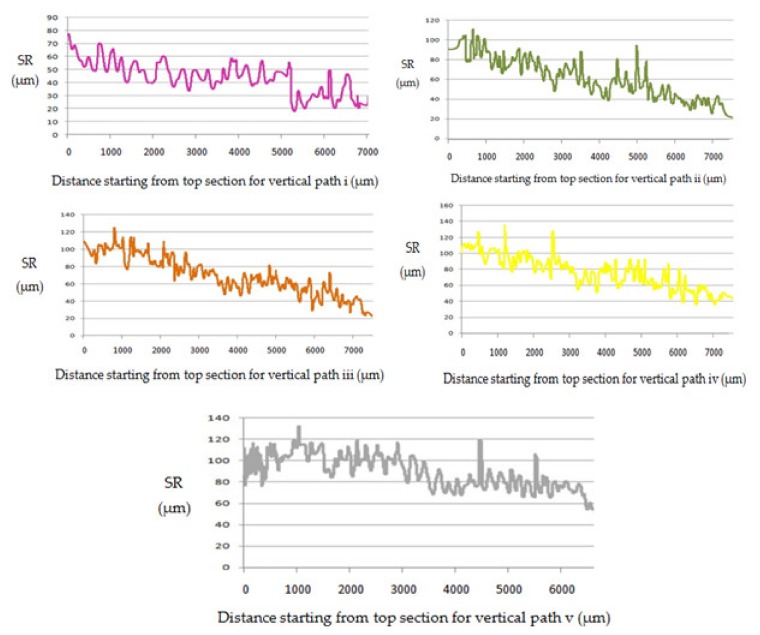
Roughness measurement along vertical plane.

The top surface of the WEDM processed sample along with SR values in µm is shown in Figure 2. Maximal surface roughness was obtained at the top-left corner of the machined surface, which is indicated by red in Figure 2. Minimal surface roughness was obtained at the bottom-right part of the machined surface, which is indicated by blue. Green on the machined surface shows the intermediate value of the surface roughness between maximal and minimal values. Figure 3a–d shows the start-to-finish points during surface-roughnessmeasurement of the machined surface, for whichthe initial point wastaken as Figure 3a; then, it wasmeasured along the periphery in a clockwise direction, as shown in Figure 3b–d. The direction of the wire travel wasas per Figure 3. DetailedSR analysis of the machined sample at different locations is presented in Figure 4. As can be seen inFigure 3, maximal SR values were obtained at the top-left corner, indicated by red. So, higherSR valueswere observed at the three outermost peripheries between points c and d. The probable reason for this is that the top surface waslargely exposed to the WEDM dielectric fluid (EDM oil) due to the side-flushing mechanism (from top), as shown in Figure 1. This resultedin faster debris removal and a higher cutting rate due to an increase in discharge-energy level. For the WEDM process, the wire-rupture problem occurred with improper settings of the machine parameters and larger craters [25]. Tosunetal. [26] investigated the effect of WEDM process parameters on crater size, and found that larger crater size increased the risk of wire rupture with increasing SR of the machined surface, along with poor machining accuracy. An increase in dielectric-fluid pressure resulted in an increase in the craters on the workpiece surface due to the high discharge-energy level [27]. This, in turn, increases work-surfaceSR. In the current study, the top surface of the workpiece was exposed to higher dielectric-fluid pressure; the SR of this region was higher as compared to that with other regions. At the bottom of the workpiece surface, dielectric supply was less, which minimized the discharge energy and improved the surface roughness of the machine surface (Figure 1). This is well-supported by the results in Figure 4. The detailed analysis for SR of the machined sample at different locations is presented in Figure 4. Five different periphery were considered for the measurement of SR. The distance considered between the two consecutive SR measurements is taken as 0.43 µm. As all the peripheries are having different circumferential lengths, number of data points for each periphery are different. For SR measurement of periphery (i), the circumferential length of periphery is 8345 µm with 19406 data points. Similarly, the circumferential length for periphery (ii), (iii), (iv) and (v) are 11810 µm, 14830 µm, 17720 µm and 20,920 µm along with the data points of 27465, 34488, 41209 and 48651 respectively. Surface roughness between the sections (c) to (d) has beem observed to be very high while in between sections (a) to (b) it is found to be on lower side. The lowest value of surface roughness was obtained between points a and b, and the highest between points c and d. Minimal and maximal peaks for the outermost periphery were obtained as 16.3 and 110.6µm, respectively. A similar trend was observed for the fourremaining peripheries, as shown in Figure 4, which shows the increase in surface finish from point a to d, with aleast surface finish of 15.25 µm, and ahighest peak of 127.67 µm. A similar trend was observed for the surface finish of the other mentioned peripheries. The minimal–maximal peak for surface roughness obtained for the third, fourth, and fifth peripheries from the outside were 24.32 and 126.26, 21.01 and 114.67, and 36.27 and 100.49 µm, respectively. These results were also validated by measuring surface roughness along the vertical plane of the machined surface of the sample machined at the same optimized parameter settings. Figure 5 shows the surface-analysis profile, showing the direction of the wire travel and path of the measurement of the surface roughness in the vertical plane. Surface roughness wasmeasured with a vertical direction at five different locations. The first vertical line from the left in Figure 5 shows that surface roughness wasmaximal as it cameintothe red section, while the last vertical line from left shows minimal surface roughness as it appeared in the blue section. These results aresupported by obtained graphs in Figure 6. The distance considered between the two consecutive SR measurements is again considered as the same with constant value of 0.43 µm. As all the five vertical lines considered for study are having different lengths, the number of data points of measurement for each vertical path are different. For SR measurement of vertical path (i), the vertical distcance is 7000 µm with 16300 data points. Similarly, the circumferential length for periphery (ii), (iii), (iv) and (v) are 11810 µm, 14830 µm, 17720 µm and 20,920 µm along with 27465, 34488, 41209 and 48651 data points respectively. A decrease in the value of surface roughness was observed, shown in Figure 5, as we proceeded for measurement from the left to the right of the machined surface. Higher surface roughness was observed at the start andlowerat the bottom. This is due to the discharge energy at the top portion, and lower discharge energy at the bottom portion of the machined surface. Thus, surface-analysis measurement along the periphery and vertical direction showed an agreement with the results. To avoid this variation in SR on the surface of a workpiece, it is recommended to have the same flushing pressure from the top and bottom of the work material. However, thismight result in an increase in average SR of the workpiece surface.

### 3.2. Scanning Morphology

**Figure 7:**
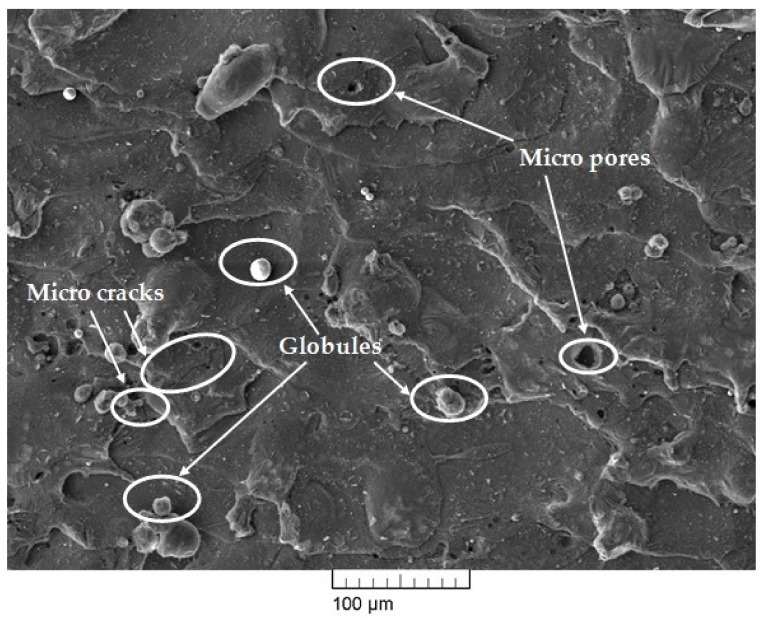
Scanning-electron-mircroscope (SEM) micrograph of machined surface obtained at high discharge-energy level.

**Figure 8:**
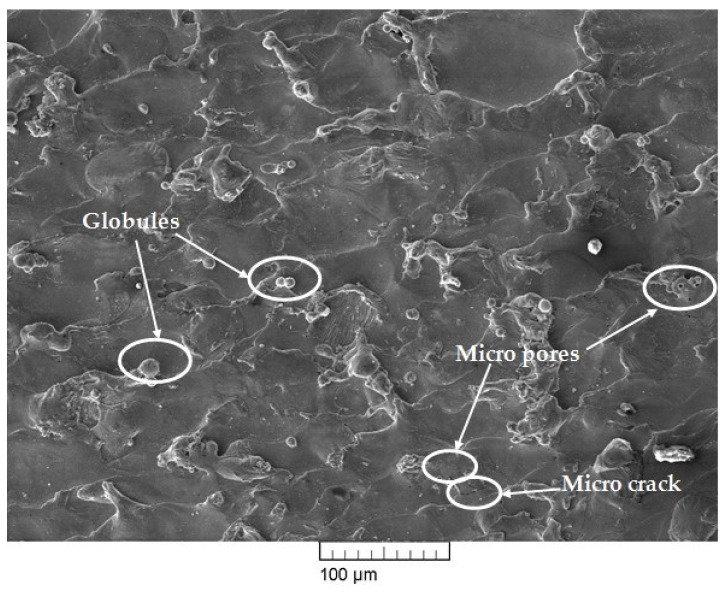
SEM micrograph of machined surface at optimized parameters.

The surface morphology of the WEDM surfaces of themachinednitinol sample is presented in Figure 7 and Figure 8. From our previous parametric-study results, it was concluded that pulse-on time, pulse-off time, and current were the most influential input-process parameters for output responses [20]. Thus, the influence of these parameters on nitinol surface integrity was studied at the optimized parameters. To satisfy multiple objectives at the same time as surface analysis, it was essential to consider the optimized parameter settings for the study. The optimized set of parameters used in the current study gavelow discharge energy, but not the lowest within the available machining-parameter range. An optimized set of parameters was determined to achieve multiresponse variables such as MRR, SR and MH. For obtaining a higher MRR, high discharge energy was required, which could be achieved with higher values of pulse-on time and current [10,25]. However, for obtaining a lower SR, low discharge energy was required thatcould be achieved with lower values of pulse-off time [10,25]. This gave the conflicting effect of input parameters on output responses such as MRR and SR. Such situations can be efficiently tackled by obtaining an optimal set of parameters for all objectives. WEDM parameter settings for the obtained optimized sample using the heat-transfer-search (HTS) algorithm had pulse-on time of 65 µs, pulse-off time of 32 µs, and discharge current of 6 A. As a result of the summation of a single spark, the machined surface was characterized by a melting zone. The surface morphology of the machined sample depended on WEDMparameter settings. High pulse-on time and high discharge current in WEDM meant high discharge energy and as a result of that a large chance of cracks, globules and other surface defects [28,29]. However, with the increase in pulse-off time, discharge energy and spark intensity decreased, which resulted in a reduction in cracks and surface defects [30]. Integrity is key in many applications in high-performance alloys [31].

**Figure 9:**
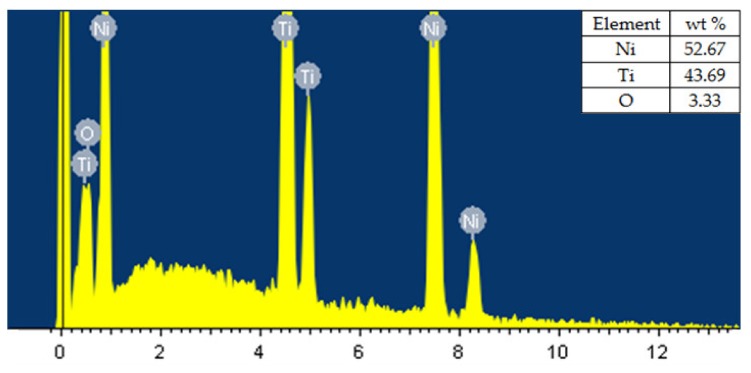
Elemental composition of nitinol shape-memory alloy(SMA) using EDX.

The parameter settings for the sample, as shown in Figure 7, hada pulse-on time of 110 µs, pulse-off time of 32 µs, and discharge current of 6 A. Machining at high pulse-on time gave high discharge energy, which resulted in large occurrences of globules, micropores, and microcracks, which can be easily seen in Figure 7. Figure 8 shows the SEM micrographs of the machined sample thatalso shows thepresence of some micropores, a deposited layer, and very few microcracks and globules. At any WEDM parameter setting, there is some amount of discharge-energy level [19]. Thus, these microcracks, deposited layers, micropores, and globules cannot be completely eliminated. However, process-parameter optimization was able to significantly reduce the deterioration of the machined surface to a large extent, as discussed in the present study. Thus, the surface results obtained at optimized parameters show significantly reduced cracks and defects, and they resulted in much better surface-integrity aspects. Elemental analysis was carried out by using energy dispersive X-ray (EDX)analysis. The researchers used a brass wire as a tool electrode, and reported that the element of the wire material was deposited on the surface of the machined surface [28,30]. Figure 9 shows the result of EDX analysis for the machined sample obtained at the optimized parameter settings. While performing EDX analysis, elements such as Ni, Ti, Mo, Cu, Al, and C were considered for elemental analysis. EDX analysis shows the presence of nickel and titanium elements of workpiece and little amount of oxygen content. The oxyden content is present due to high temperature induced during machining and high activity of Ti and Ni atoms. Absence of Mo in EDX analysis means it shows no deposition of wire material on the work surface. This can be considered as one of the notable outcomes of the present study. However with the use of reusable Mo wire, it was observed that, even at higher discharge energies, the wire material was not deposited on the workpiece material. This may have been due to the capabilities of Mo wire to withstand aggravated chemical reactions between anode, cathode, and dielectric fluid. One of the inherent benefits of using higher discharge energies is that higher MRR can be achieved, increasing productivity.

## 4. Conclusions

The present study focused on analyzing the surface integrity of a WEDM sample obtained at optimized parameter settings. Resultsindicated that, during the WEDM of nitinol SMA, appropriate discharge energy is required to achieve optimalSR. Greater discharge energy at the surface in contact with the wire showed increased surface roughness in areas where EDM oil was used in a good amount during machining. The selected parametersat optimized conditions (pulse-on time 65 µs, pulse-off time 32 µs, and discharge current 6 A) indicated a defectfree surface, along with a decrease inthe size and number of globules. The selected parameters were able to machine the alloy without wire erosion, which was formulated from EDX analyses. EDX analysis shows the presence of nickel and titanium elements of workpiece and little amount of oxygen. Work surface has been observed without the deposition of wire material. This result showed the suitability of the parameters and wire material (molybdenum) for the machining of nickel–titanium shape-memory alloys.

## Figures and Tables

**Table 1:** Nitinolcomposition (wt%).

Element	Ti	Ni	Co	Cu	Cr	Fe	Nb	C	H	O	N
wt%	Balance	55.78	0.005	0.005	0.005	0.012	0.005	0.04	0.001	0.035	0.001

## Nomenclature

A	Ampere
EDX	Energy dispersive x-ray
HTS	Heat transfer search
MH	Microhardness (HV)
MOHTS	Multi-objective heat transfer search
MRR	Material removal rate (mm^3^/sec)
SEM	Scanning electron microscopy
SMA	Shape memory alloy
SMAs	Shape memory alloys
SR	Surface roughness (µm)
T_on_	Pulse on time (µs)
T_off_	Pulse off time (µs)
WEDM	Wire electric discharge machine
WEDMed	Wire electric discharge machined
